# They are not real patients

**DOI:** 10.1192/j.eurpsy.2021.1823

**Published:** 2021-08-13

**Authors:** T. Jiménez Aparicio, C. De Andrés Lobo, C. Vallecillo Adame, M. Queipo De Llano De La Viuda, G. Guerra Valera, A. Gonzaga Ramírez, J. Gonçalves Cerejeira, I. Santos Carrasco, C. Capella Meseguer, E. Rodríguez Vázquez

**Affiliations:** 1 Psiquiatría, Hospital Clínico Universitario Valladolid, Valladolid, Spain; 2 Psiquiatría, HCUV, Valladolid, Spain; 3 Psiquiatría, Hospital Clínico Universitario Valldolid, Valladolid, Spain; 4 Psiquiatría, Hospital Clínico Universitario de Valladolid, Valladolid, Spain

**Keywords:** Depressive pseudodementia, psychotic depression, cognitive impairment, cognitive depressive disorder

## Abstract

**Introduction:**

Cognitive depressive disorder (or depressive pseudodementia) is a condition defined by functional impairment, similar to dementias or other neurodegenerative disorders, in the context of psychiatric patients. It is important to consider a differential diagnosis in patients with cognitive impairment.

**Objectives:**

Presentation of a clinical case of a patient with depression with psychotic symptoms who presents cognitive impairment.

**Methods:**

Bibliographic review of the differential diagnosis between cognitive depressive disorder and real dementia by searching for articles in PubMed.

**Results:**

We present a 51-year-old woman, previously diagnosed with adjustment disorder (with mixed anxiety and depressed mood) and unspecific anxiety disorder, who was admitted to the hospital due to delusional ideation of harm and Capgras syndrome, ensuring that her relatives had been replaced and the rest of the patients were not real patients, but actors who conspired against her. The MRI (Magnetic Resonance Imaging) was strictly normal (tumors or acute injuries as stroke or hemorrhage were discarded), and a MoCA (Montreal Cognitive Assesment) test was performed to screen any cognitive impairments (obtaining a score of 19/30, with language fluency and abstraction particularly affected). It would be convenient to repeat the test when this episode and the psychotic symptoms are resolved or improved.

**Conclusions:**

1. Some patients may have cognitive impairment in the context of a mood disorder. 2. A differential diagnosis and follow-up of these patients should be performed to assess prognosis, reversibility and treatment. 3. Depressive cognitive impairment may precede the development and establishment of a dementia or neurodegenerative picture.

**Disclosure:**

No significant relationships.

